# Diversification in wild populations of the model organism *Anolis carolinensis*: A genome‐wide phylogeographic investigation

**DOI:** 10.1002/ece3.2547

**Published:** 2016-10-17

**Authors:** Joseph D. Manthey, Marc Tollis, Alan R. Lemmon, Emily Moriarty Lemmon, Stéphane Boissinot

**Affiliations:** ^1^ New York University Abu DhabiAbu DhabiUAE; ^2^ Biodesign InstituteArizona State UniversityTempeAZUSA; ^3^ Department of Scientific ComputingFlorida State UniversityTallahasseeFLUSA; ^4^ Department of Biological ScienceFlorida State UniversityTallahasseeFLUSA

**Keywords:** Anolis, phylogeography, species tree, target capture

## Abstract

The green anole (*Anolis carolinensis*) is a lizard widespread throughout the southeastern United States and is a model organism for the study of reproductive behavior, physiology, neural biology, and genomics. Previous phylogeographic studies of *A. carolinensis* using mitochondrial DNA and small numbers of nuclear loci identified conflicting and poorly supported relationships among geographically structured clades; these inconsistencies preclude confident use of *A. carolinensis* evolutionary history in association with morphological, physiological, or reproductive biology studies among sampling localities and necessitate increased effort to resolve evolutionary relationships among natural populations. Here, we used anchored hybrid enrichment of hundreds of genetic markers across the genome of *A. carolinensis* and identified five strongly supported phylogeographic groups. Using multiple analyses, we produced a fully resolved species tree, investigated relative support for each lineage across all gene trees, and identified mito‐nuclear discordance when comparing our results to previous studies. We found fixed differences in only one clade—southern Florida restricted to the Everglades region—while most polymorphisms were shared between lineages. The southern Florida group likely diverged from other populations during the Pliocene, with all other diversification during the Pleistocene. Multiple lines of support, including phylogenetic relationships, a latitudinal gradient in genetic diversity, and relatively more stable long‐term population sizes in southern phylogeographic groups, indicate that diversification in *A. carolinensis* occurred northward from southern Florida.

## 1 Introduction

A current necessity in evolutionary biology is to understand how evolutionary history shapes natural variation in model organisms for complex traits (Gasch, Payseur, & Pool, 2016). The green anole lizard (*Anolis carolinensis*) was the first nonavian reptile to have a complete genome sequence (Alföldi et al., 2011) and is an indispensable laboratory model for biomedical fields such as reproductive endocrinology (Lovern, Holmes, & Wade, 2004; Wade, 2012) and appendage regeneration (Hutchins et al., 2014). However, unlike studies using established models such as the house mouse (*Mus musculus*), which rely on inbred strains, green anole laboratory protocols are based on wild‐caught individuals. This is despite the fact that with a natural range across the southeastern United States, *A. carolinensis* exhibits wide geographic variation in morphology (Jaffe, Campbell‐Staton, & Losos, 2016) and physiology (Goodman et al., 2013), and the connection between genetic and phenotypic diversity in the species remains unknown.

More generally, the distribution of *A. carolinensis* overlaps with a suite of species with phylogeographic structure in the southeastern United States (for a review, see Soltis et al., 2006). In this region, terrestrial species’ genetic structure generally coincides with barriers such as the Appalachian Mountains and several large river systems. In many of these taxa, genetic structure was hypothesized to be a consequence of divergence in allopatry during the Last Glacial Maximum followed by subsequent range expansions out of refugia (Soltis et al., 2006). In this context, resolving the phylogeographic history of *A. carolinensis* would provide an additional reference to the biogeographic history of this region. Therefore, in order to better develop *A. carolinensis* as a model in biomedical and genomic research, as well as compare its evolutionary history with broader biogeographic patterns, a clear picture of its phylogeographic and demographic history is necessary.

The evolutionary history of *A. carolinensis* has yet to be fully resolved, due to differing conclusions that are based on only a few genetic markers. The species is phylogenetically nested within the Cuban green anole *A. porcatus*, and there is agreement that it originated in Florida after overwater dispersal from Cuba (Buth, Gorman, & Leib, 1980; Glor, Losos, & Larson, 2005). Recent analyses of mitochondrial DNA (mtDNA) fragments and small numbers of nuclear DNA loci agree that Florida contains most of green anole genetic diversity, and the intrapopulational distributions of DNA polymorphisms suggest population size expansions on the continental mainland (Campbell‐Staton et al., 2012; Tollis, Ausubel, Ghimire, & Boissinot, 2012; Tollis & Boissinot, 2014). Based on these conclusions, it was suggested that early green anole divergence was fueled by vicariance across Pleistocene island refugia on the Florida peninsula, followed by more recent dispersal both northwards along the Atlantic seaboard and west across the Gulf Coastal Plain (Tollis & Boissinot, 2014).

**Figure 1:**
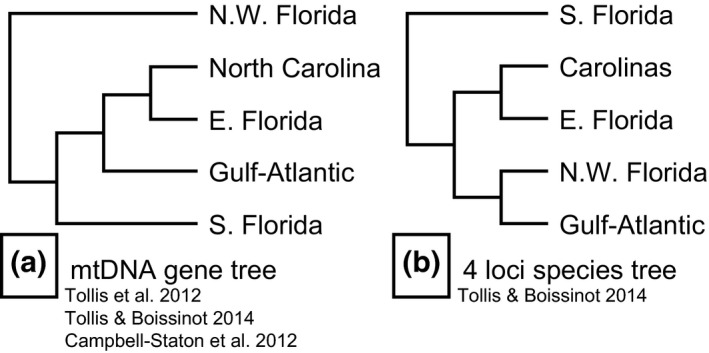
Phylogenies estimated from previous studies, simplified to the five main genetic clusters for clarity. Group names refer to the same regions and genetic clusters as shown in Figures 2, 3, 4, 5, with the exception of the Carolinas. In the mtDNA studies (a), South Carolina was in the Gulf‐Atlantic clade, while North Carolina had its own clade. In the multilocus tree (b), the Carolinas clade was mostly North Carolina, with some individuals of South Carolina

Previous phylogeographic analyses of *A. carolinensis* identified five geographically structured clades across the species range: three in Florida and two out of Florida (Campbell‐Staton et al., 2012; Tollis & Boissinot, 2014; Tollis et al., 2012). However, these studies identified conflicting and poorly supported relationships among the clades. All three studies found a sister relationship between localities in the Carolinas (North and South) and eastern Florida (Figure 1). Phylogenies based on mtDNA identified western and northwestern localities in Florida as sister to all other populations (Figure 1a; Campbell‐Staton et al., 2012; Tollis et al., 2012), while southern Florida (i.e., Everglades) localities were sister to all other populations using a species tree analysis (Figure 1b, Tollis & Boissinot, 2014). In both trees (Figures 1a,b), all but two clades had different sister relationships. Thus, the branching order of divergence events and true relatedness of subpopulations within *A. carolinensis* remain unresolved, obscuring the potential effects of evolutionary history on biomedical studies that may include green anoles from different sampling localities. Therefore, increased effort to resolve relationships between *A. carolinensis* subpopulations with a larger sampling of genetic loci is needed.

Recently developed methodologies such as restriction site‐associated DNA sequencing (RAD‐seq, Miller et al., 2007) and target capture using ultraconserved elements (UCEs, Faircloth et al., 2012) or anchored hybrid enrichment (AHE, Lemmon, Emme, & Lemmon, 2012) now allow researchers to obtain reduced representation genomic coverage across many individuals. All three types of data collection have been shown to be appropriate for phylogeographic‐level studies of vertebrates, including RAD‐seq (Manthey & Moyle, 2015), UCEs (Smith et al., 2013), and AHE (Brandley et al., 2015), suggesting these methods’ ability to resolve the evolutionary history of *A. carolinensis*. Here, we used more than 500 genome‐wide loci collected via AHE with the following goals: (1) clarify the evolutionary relationships of previously identified clades in *A. carolinensis*, (2) explore patterns and trends of genetic diversity and differentiation within and among lineages, (3) elucidate the demographic history and timing of diversification within the species, and (4) compare the phylogeographic patterns found in *A. carolinensis* with other species from the southeastern United States.

## 2 Methods

### 2.1 Sampling and laboratory procedures

**Figure 2:**
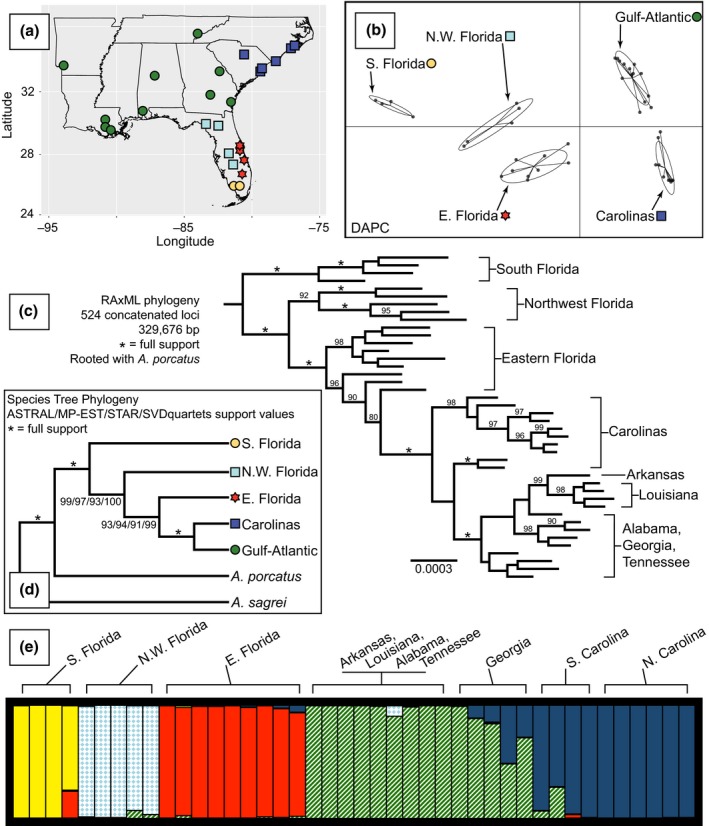
Sampling and genetic structure. (a) Sampling localities of *Anolis carolinensis* in the U.S. Southeast. Symbols match corresponding genetic clusters from DAPC analyses. (b) Five genetic clusters identified from DAPC analyses. (c) RAxML phylogenetic tree from 524 concatenated loci. Nodes with <70% bootstrap support are unlabeled. (d) Results of species tree analyses. ASTRAL, MP‐EST, and STAR utilized gene trees generated from loci containing ≥5 parsimony informative sites. All loci were concatenated for use in the SVDquartets analysis. (e) STRUCTURE analysis result for one of the SNP datasets. Each vertical bar represents one individual (proportion of color informs inferred probability of population ancestry)

We sampled 42 *A. carolinensis* individuals from 26 localities across its distributional range (Figure 2a; Table S1) encompassing the five major clades identified in previous molecular work (Campbell‐Staton et al., 2012; Tollis & Boissinot, 2014; Tollis et al., 2012). Individuals of both *A. porcatus* and *A. sagrei* were used as outgroups. All samples were collected for previous studies (Tollis & Boissinot, 2014; Tollis et al., 2012). For the current sequencing experiment, genomic DNA was extracted via proteinase K digestion followed by purification with the Promega Wizard Genomic DNA Purification standard protocol and elution in TE buffer. DNA samples were quantitated using a Nanodrop Spectrophotometer to ensure a 260/280 absorbance ratio of 1.8 or above and were precipitated in ethanol to a concentration ≥20 ng/μl.

Data were collected following the general methods of Lemmon et al. (2012) through the Center for Anchored Phylogenomics at Florida State University (www.anchoredphylogeny.com). Each genomic DNA sample was sonicated to a fragment size of ~300–800 bp using a Covaris E220 Focused‐ultrasonicator with Covaris microTUBES. Following a protocol modified from Meyer and Kircher (2010), library preparation and indexing were performed on a Beckman Coulter Biomek FXp liquid‐handling robot. One modification consists of a size‐selection step after blunt‐end repair using SPRIselect beads (Beckman Coulter Inc.; 0.9× ratio of bead to sample volume). Indexed samples were pooled at equal quantities (12–16 samples per pool), and enrichments were performed on each pool using an Agilent Custom SureSelect kit (Agilent Technologies), which contained probes designed for anchored loci from multiple vertebrate genomes (Vertebrate v1 probe design; Lemmon et al., 2012). After enrichment, the three enrichment reactions were pooled in equal quantities for sequencing on PE150 Illumina HiSeq2000 lanes. Sequencing was performed in the translational science laboratory in the College of Medicine at Florida State University.

### 2.2 Bioinformatics

Following Prum et al. (2015), reads passing the high chastity CASAVA filter were assembled as follows. After merging overlapping reads (Rokyta, Lemmon, Margres, & Aronow, 2012), reads were assembled using *A. carolinensis* references derived from the Vertebrate v1 probe design (Lemmon et al., 2012). Resulting consensus sequences were phased in a Bayesian framework using reads overlapping with polymorphic sites, as described by Pyron et al. (2016). Orthology was assessed using sequence similarity (see Prum et al. for details), and orthologous sequences were aligned using MAFFT (v7.023b Katoh & Standley, 2013) and then trimmed/masked to remove ambiguously aligned regions. Methodological details and scripts are provided in Prum et al. (2015) and Pyron et al. (2016).

We matched all loci to the *A. carolinensis* genome (AnoCar2.0) using Megablast (Zhang, Schwartz, Wagner, & Miller, 2000) implemented in the database resources of the National Center for Biotechnology (NCBI; Wheeler et al., 2003). For each locus, we estimated the average number of pairwise differences between individuals within clades (π; see 3) and an estimate of genetic differentiation (Hudson, Slatkin, & Maddison, 1992) between clades. Lastly, we used R (R Development Core Team 2012) to identify fixed, shared, and private single nucleotide polymorphisms (SNPs) within phylogeographic groups based on the sequences of the phased alleles for each locus.

### 2.3 Estimating genetic structure using single nucleotide polymorphisms

We used two methods utilizing SNPs extracted from the target capture loci to assess genetic structure between *A. carolinensis* samples. First, we used discriminant analysis of principal components (DAPC; Jombart, Devillard, & Balloux, 2010), implemented in the R adegenet package (Jombart & Ahmed, 2011). DAPC sequentially uses principal components of SNP data followed by discriminant analysis to ascertain genetic groupings. We used spline interpolation (Hazewinkel, 1994) to identify the appropriate number of principal components to retain in the discriminant analysis; we retained two principal components and two of the linear discriminants (Table S3).

Next, we used the program STRUCTURE (Pritchard, Stephens, & Donnelly, 2000) to further explore phylogeographic structure in the data. We created two datasets that each selected a random SNP per target capture locus. With these two datasets, we performed the following methodologies twice. First, we used an initial run to infer lambda while estimating the likelihood of one population (*k* = 1; Pritchard et al., 2000). Using the inferred value of lambda, we ran structure estimating the likelihood of between one and nine genetic clusters (*k* = 1–9; five replicates each) using the admixture model and correlated allele frequencies. We ran the analyses for a burn‐in of 50,000 steps followed by 50,000 MCMC iterations. Lastly, we used the Δ*K* method of Evanno, Regnaut, and Goudet (2005) to identify the number of genetic clusters from the STRUCTURE output.

### 2.4 Phylogenetic analyses

Using all loci in a concatenated data matrix, we estimated the phylogenetic relationships of all individuals using RAxML (Stamatakis, 2014), using a GTR + Gamma model of sequence evolution as estimated by model selection implemented in PAUP* v4.0a147 (Swofford, 2003). Here, we used the *A. porcatus* sample to root the phylogeny. We limited gene trees for species tree analysis to include only those with five or more parsimony informative sites and sampled in *A. sagrei* (273 loci), as gene trees based on little or no informative sites may negatively impact species tree analyses (Manthey, Campillo, Burns, & Moyle, 2016). We estimated gene trees of all loci using RAxML (GTR + Gamma model of sequence evolution). For the 273 loci, we created 100 multilocus bootstraps (Seo, 2008) that resample both loci within the dataset and bases within a locus. We used three methods of species tree inference: (1) accurate species tree algorithm (ASTRAL; Mirarab et al., 2014), (2) maximum pseudo‐likelihood of estimating species trees (MP‐EST; Liu, Yu, & Edwards, 2010), and (3) species trees from average ranks of coalescence (STAR; Liu et al., 2009). In these analyses, the *A. sagrei* sample designated the outgroup. We used the 273 loci dataset to estimate the species trees, with the multilocus bootstrap replicates to assess support of nodes within the species trees.

With the gene trees generated in RAxML, we also calculated the genealogical sorting index (GSI; Cummings, Neel, & Shaw, 2008). The GSI uses gene trees to measure exclusive ancestry of predefined groups and can be directly compared across loci. GSI values range from zero to one, indicating the continuum from random mixing of a group's individuals across a gene tree (GSI = 0) to monophyly of a group (GSI = 1). In addition to per locus GSI estimates, we calculated the ensemble GSI (GSI_T_), a summary statistic of all gene trees, that is calculated using the weighted sum of the GSI values from each tree topology (Cummings et al., 2008).

In addition to the GSI tests across loci, we also investigated phylogenetic discordance across gene trees using BUCKy v1.4.4 (Larget et al. 2010). Posterior distributions of gene trees are used as the input of BUCKy, and necessarily have no missing data and small sample sizes of tips (Larget et al. 2010). For this reason, we selected all highly informative loci used in previous gene tree analyses without missing data (*n* = 217 loci). From these loci, we created two datasets where we randomly selected one allele from each lineage of *A. carolinensis* (see 3), including the outgroup *A. porcatus*. We generated posterior distributions of gene trees using MrBayes v3.2 (Ronquist et al. 2012). In MrBayes, the MCMC was run for ten million generations and sampled every 10,000. The posterior distributions of trees were summarized using the *mbsum* function implemented in BUCKy, where we included the final 100 trees of each MrBayes run for input in BUCKy. In BUCKy, we used the default parameters, while varying the value of α (α = 10, 100, 1,000). The α parameter represents the expected amount of discordance across loci.

We estimated a species tree independent of gene trees using SVDquartets (Chifman & Kubatko, 2014). SVDquartets infers topologies of quartets of individuals in a coalescent framework and then uses those topologies to create a species tree. We sampled 100,000 quartets from the dataset and estimated confidence in the topology with 100 bootstrap replicates. Finally, we used TreeMix (Pickrell & Pritchard, 2012), which utilizes SNPs and incorporates migration events into the phylogeny. Initially, TreeMix infers a maximum‐likelihood species phylogeny, followed by linking species or populations with candidate migration events when they are more closely related than can be explained by the species tree (Pickrell & Pritchard, 2012). We ran TreeMix with all SNPs pulled from all loci and performed 100 bootstraps replicates to assess confidence in phylogeny estimation using 200 SNP bootstrap blocks. We added migration edges until they explained >99.8% of the variance in the SNP data (Pickrell & Pritchard, 2012); this resulted in one migration edge. To assess whether SNP linkage impacted TreeMix results, we created two additional datasets. Here, we used TreeMix with two datasets, each containing one randomly sampled SNP per locus.

### 2.5 Demographic analyses

Because the loci used here flank conserved regions across multiple vertebrate genomes (Lemmon et al., 2012), some signatures of nearby genomic purifying selection may be present that could impact demographic analyses. In a recent comparison of RAD‐seq and target capture for demographic analyses, Harvey et al. (2016) identified similar estimates of theta (θ = 4N_e_μ) among marker types within populations, but relatively different estimates when extended to inferring ancestral population sizes. This comparison of RAD‐seq (a putatively neutral genomic marker set) and target capture (possibly linked to sites under purifying selection) suggests demographic results within a population or lineage, but not extending back to ancestral populations with multiple lineages, are robust when limited to comparing populations’ relative demographic estimates.

To infer the history of each of the five phylogeographic clusters (see 3), we estimated extended Bayesian skyline plots (EBSPs; Heled & Drummond, 2008) in BEAST 2.3.2 (Bouckaert et al., 2014) for each of the five phylogeographic clusters to infer their demographic histories. We used previously published mtDNA data (Tollis & Boissinot, 2014; Tollis et al., 2012) and the thirty most informative loci using prior and operator setups recommended for large datasets (Trucchi et al., 2014). We enforced a strict clock on the mtDNA (divergence rate mean = 0.013, corresponding to 0.065 changes/site/million years in each lineage, range = 0.005–0.008), a widely used estimate for iguanian lizards (Macey et al., 1998), with all other loci evolving clocklike relative to mtDNA. We used a strict clock due to the intraspecific nature of the investigation and to avoid overparameterization from more complex clock models with the large number of genetic loci. Models of sequence evolution were estimated for each of the loci using model selection implemented in PAUP* v4.0a147 (Swofford, 2003) and chosen using a Bayesian information criterion. BEAST was run for one billion generations, with the first 50% used as burn‐in. Appropriate mixing and effective sample sizes of all parameter estimates were visualized using TRACER, a program implemented with BEAST.

To obtain a secondary estimate of effective population sizes, as well as identify relative divergence timing, we used G‐PhoCS (Gronau et al., 2011). We used the recommended settings for the program that had been utilized in recent phylogeographic datasets (Campagna et al., 2015). Because it was not computationally possible to run the program with all individuals, we ran the program with three datasets, each using three individuals sampled from each clade (as in Figure 2) and one *A. porcatus* individual. Initially, we attempted to incorporate migration bands between clades, but were unable to obtain convergence on parameter estimates. Therefore, we continued only estimating relative divergence times and population sizes. For each dataset, we ran the program for 504,899 MCMC steps (5,000 samples), skipping 100 steps between samples, and removing the first 20% of iterations as burn‐in.

## 3 Results

### 3.1 Target capture and sequencing

The number of sequencing reads per individual was highly variable, ranging from ~3.8 million to ~20.3 million (Table 1, Table S1). The number of targeted loci recovered across individuals was consistently around 500 per individual of a total of 524 (Table 1). Contig length was also variable, ranging from 336 to 887 bp (Table 1), with different numbers of parsimony informative characters (range 0–26; Table 1). The mean sequencing coverage for all loci within individuals was high (mean = 2547 reads, SD = 595; Table 1; Table S1).

**Table 1:** Sequencing data and genetic loci summary statistics. All values (except percent missing data) rounded to nearest integer. Per individual statistics are summarized in Table S1

	Mean ± SD	Minimum	Maximum
Sequence reads	8,883,159 ± 2,858,211	3,769,666	20,314,656
No. of loci^a^	508 ± 4	487	512
Average locus length^a^	716 ± 56	539	842
Average reads per locus^a^	2547 ± 595	1,029	4,781
% Missing loci^a^	3.06 ± 0.76	2.29	7.06
Average contig length (bp)^b^	629 ± 70	336	887
SNPs per locus^b^	17 ± 8	3	56
PI SNPs per locus^b^	6 ± 4	0	26

PI, Parsimony Informative; SNP, single nucleotide polymorphism.

a Summary statistics using each individual's reads and locus distribution information.

b Statistics summarizing each contig locus used in phylogenetic analyses.

### 3.2 Phylogeographic structure

**Figure 3:**
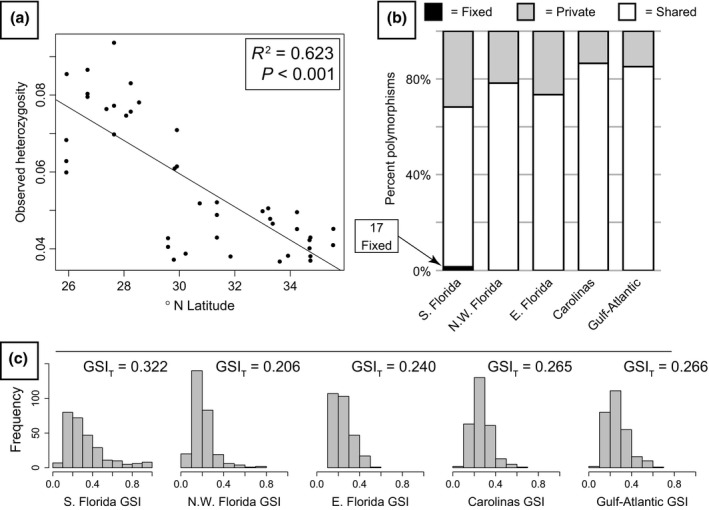
Genetic diversity and genealogical sorting. (a) Relationship of observed heterozygosity and latitude for all *Anolis carolinensis* individuals. (b) Proportion of fixed, shared, and private polymorphisms for each genetic cluster, excluding singletons (variants only found as heterozygous in one individual). (c) Genealogical sorting index (GSI) histograms for each genetic cluster across 273 gene trees used in species tree analyses (Fig. 2d) and the ensemble GSI (GSI_T_) using information from all trees

Utilizing ~9,000 SNPs from 524 loci, the DAPC analysis indicated five genetic clusters (Figure 2b). These five groups correspond to the five clades identified in previous phylogenetic analyses (Campbell‐Staton et al., 2012; Tollis & Boissinot, 2014; Tollis et al., 2012) and were used as a priori groupings for other analyses investigating diversity, differentiation, and phylogenetics. In these five genetic clusters, the southern Florida group was the only one with fixed differences (17; Figure 3b, Table 2). Generally, the more southern clades had a higher proportion of private relative to shared polymorphisms (Figure 3b, Table 2). Within each of the phylogeographic clusters, patterns of genetic diversity and differentiation showed no obvious patterns across chromosomes (Table S4). As with DAPC, in STRUCTURE, we identified five genetic clusters using the Δ*K* method of Evanno et al. (2005; Table S2). The two STRUCTURE analyses identified very similar results (*R* ^2^ = .981 of assignment probabilities), with generally strong genetic structure in Florida (Figure 2e, Table S2), and some signal of admixture between the Gulf‐Atlantic and Carolinas phylogeographic clusters.

**Table 2:** Summary of fixed, shared, and private polymorphisms in the five clades of *Anolis carolinensis*, including singletons

Clade	Fixed	Shared	Private
South Florida	17	808	1,147
Northwest Florida	0	1,293	1,222
Eastern Florida	0	1,581	2,049
Carolinas	0	946	815
Gulf‐Atlantic	0	1,130	1,479

We used all 524 loci in a concatenated matrix for phylogenetic analysis in RAxML; here, we identified three of the DAPC‐identified clusters (south Florida, northwest Florida, and Gulf‐Atlantic) as monophyletic, with the other two paraphyletic (Figure 2c). Based on GSI results, monophyly of each of the five phylogeographic groups in gene trees is the exception and not the rule (Figure 3c), with GSI distributions of each clade centered between values of 0.2 and 0.4. These distributions were reflected in the GSI_T_ values, where all clades identified using DAPC had significant (*p* < .01) but moderate values of GSI_T_ (range: 0.206–0.322) when considering all analyzed gene trees (Figure 3c). The southern Florida clade was the only group with GSI values reaching one (Figure 3c).

**Figure 4:**
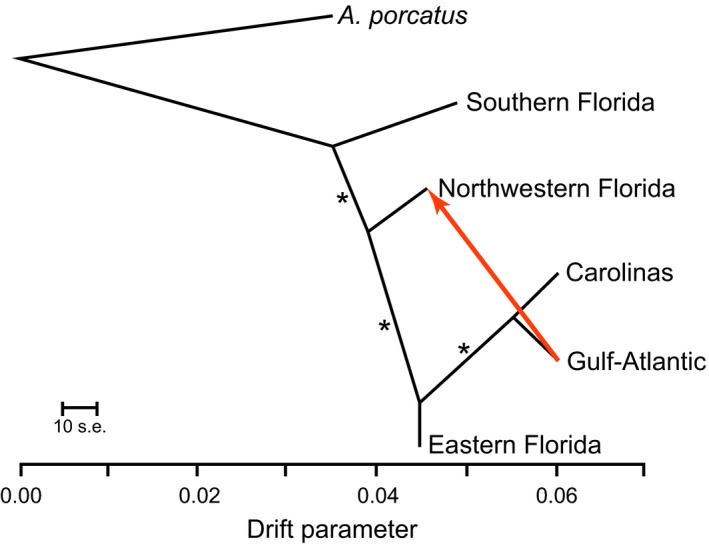
Output of Treemix analysis, identifying the same relationships as species tree analyses (Fig. 2d). One gene flow event was inferred between the Northwestern Florida and Gulf‐Atlantic phylogeographic clusters. With no migration events, the species tree explains 99.6% of the variation in the SNP data, with an additional 0.3% explained by the potential migration event. Asterisks indicate full support from 100 bootstraps

Using all highly informative (5 + PI sites) gene trees in three species tree analyses, we identified strongly supported relationships that are novel relative to previous work (Figure 2d). The northernmost groups (Gulf‐Atlantic and Carolinas) were most closely related and were found sister to eastern Florida (Figure 2d). This grouping of eastern Florida and Gulf‐Atlantic + Carolinas was sister to the northwestern Florida clade. The southern Florida clade was identified as sister to all other *A. carolinensis* groups (Figure 2d). TreeMix analyses identified the same species tree as all other species tree analyses (Figure 4), with one inferred gene flow event between contemporary populations of the northwestern Florida and Gulf‐Atlantic phylogeographic clusters. TreeMix analyses (two replicates) limited to one random SNP per locus identified the same topology as the full dataset. One replicate included no migration edges, and the second identified the same migration edge as the full dataset (Table S8). The SVDquartets analysis identified the same species tree as other analyses (Figure 2d).

In BUCKy, using different values of α resulted in the same primary concordance tree topologies within a dataset. However, using different subsamples of alleles from different lineages resulted in different primary concordance tree topologies (i.e., different relationships among lineages; Table S9). There were two consistencies across data subsets used in BUCKy: (1) The southern Florida group was basal to all other *A. carolinensis* lineages, and (2) the Carolinas and Gulf‐Atlantic clades were sister. The relationships of the eastern and northwestern Florida lineages varied across the data subsets. Even with the consistencies across BUCKy analyses, the ranges of concordance factors (0.152–0.673; Table S9) suggest a general trend of incomplete lineage sorting across most loci. The inability to use all individuals in BUCKy—due to the massive increase in gene tree space as number of tips increases—potentially limited our results in this analysis and may have been compounded by the nature of the genetic markers used and the recent nature of divergence between the *A. carolinensis* lineages.

### 3.3 Demographic and divergence timing analyses

**Figure 5:**
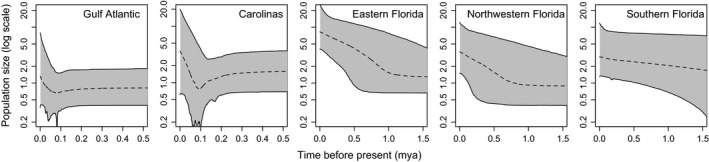
Extended Bayesian skyline plots for each of the five genetic clusters. The dotted line is the median estimate, with the 95% highest posterior density shaded. Note the *x*‐axis varies between plots

Based on EBSPs, all three Florida groups appear to have undergone steady population size increases over the last 0.25–1.5 million years (my, Figure 5). In contrast, the Carolinas and Gulf‐Atlantic groups each showed a short population decline between 0.05 and 0.12 million years ago (mya) followed by sharp increases in population sizes (Figure 5). Current population sizes were highest in eastern Florida and lowest in the Gulf‐Atlantic clade, relative to other groups (Figure 5). To further investigate demographic patterns, we looked at the relationship between genetic diversity and latitude. We might expect more stable populations or origins of diversification (i.e., ancestral population locations) to have higher genetic diversity relative to recently colonized or less stable populations. Here, we identified a negative relationship between latitude and genetic diversity (observed heterozygosity of SNPs) across all individuals (Figure 3a).

The G‐PhoCS analyses showed consistent results even when using different sets of individuals from each genetic cluster (Table 3). Population sizes estimated in G‐PhoCS were positively correlated with current population sizes from BEAST EBSP analyses, although the relationships were not significant (.05 < *p* < .20; Table S5). Population sizes estimated with different individuals’ data showed consistent estimates (all *R* ^2^ > .9 and *p* < .05; Table S6). Because G‐PhoCS outputs relative divergence times, we used multiple previous estimates of divergence dates between western *A. porcatus* and *A. carolinensis* to estimate timing of diversification within *A. carolinensis*. In other words, the root divergence between *A. porcatus* and *A. carolinensis* was calibrated using three previously published divergence dates between the two species, with all intraspecific splits estimated relative to the calibrated root divergence. First, we used 12.3 mya as the root divergence date, the central estimate of Campbell‐Staton et al. (2012), where they used a relaxed molecular clock of the ND2 mtDNA gene and a clock rate of 1.3% pairwise divergence per million years. Second, we used the uncorrected pairwise divergence between western *A. porcatus* and *A. carolinensis* ND2 sequences (0.087) from Glor et al. (2005) and a 1.3% clock rate, which gives an estimate of ~6.7 mya. The uncorrected distances were used here to include the whole gamut of possible divergence times, as the corrected distances would produce a result nearly identical to the strict clock of Tollis et al. (2012). Lastly, we used the median estimate (~6.2 mya) of Tollis et al. (2012), who again used ND2 and a strict molecular clock.

**Table 3:** Divergence time estimates. Results of relative divergence time estimates from G‐PhoCS analyses, including the median and 95% HPD interval. Other columns indicate a range of divergence time estimates (in bold) calibrated from different datasets (Tollis, Glor, Campbell‐Staton) and methodologies (see 3). All other values—within *Anolis carolinensis*—are based on these calibrations and the relative divergence estimates (median) from G‐PhoCS. All G‐PhoCS values are relative estimates, and all other values are in millions of years before present

Divergence	G‐PhoCS	Tollis (2012)	Glor (2005)	Campbell‐Staton (2012)
1. Gulf‐Atl, Carolinas	2.01 (1.51–2.66)	0.97 (0.79–1.18)	1.05 (0.86–1.28)	1.92 (1.57–2.35)
2. Group 1 + E. Florida	2.78 (2.30–3.39)	1.34 (1.21–1.51)	1.45 (1.31–1.63)	2.66 (2.40–2.99)
3. Group 2 + N.W. Florida	2.80 (2.32–3.41)	1.35 (1.22–1.52)	1.46 (1.32–1.64)	2.68 (2.42–3.01)
4. Group 3 + S. Florida	6.64 (5.88–7.31)	3.20 (3.09–3.25)	3.46 (3.34–3.52)	6.35 (6.13–6.45)
5. *Anolis porcatus* + *Anolis carolinensis*	12.87 (11.80–13.93)	**6.2**	**6.7**	**12.3**

Although these datasets and estimates are all based on the same mtDNA gene, previously estimated divergence times between *A. carolinensis* and *A. porcatus* vary widely based on different molecular clock models. Because of this, we use all of these calibrations cautiously and only in an attempt to identify general time frames of divergence between major *A. carolinensis* phylogeographic clusters. Based on median relative divergence estimates from G‐PhoCS analyses (Table 3), the southern Florida clade diverged from all other *A. carolinensis* in the late Miocene or Pliocene. All other divergence events are estimated to have occurred during the Pleistocene, or possibly the late Pliocene, in rapid succession (Table 3).

## 4 Discussion

We sequenced hundreds of loci for 42 *A. carolinensis* individuals sampled across the species’ distribution, resolved the evolutionary history of five phylogeographic clusters, and identified mito‐nuclear discordance when comparing our results to previous studies (Campbell‐Staton et al., 2012; Tollis & Boissinot, 2014; Tollis et al., 2012). The general direction of diversification in *A. carolinensis* appears to be northward based on multiple lines of support: (1) Phylogenetic estimates (Figures 2 and 4) indicate a step‐wise diversification pattern out of southern Florida, (2) genetic diversity shows a latitudinal cline (Figure 3a), including more private polymorphisms in the south (Figure 3b), and (3) southern populations have been stable longer with a relatively constant growth, while northern populations had recent population size decreases followed by rapid expansion (Figure 5).

### 4.1 Mito‐nuclear discordance among phylogeographic clades

This study adds to the growing number of papers identifying mito‐nuclear discord (for a review, see Toews & Brelsford, 2012). Using hundreds of loci, we found the same phylogeographic clusters—some paraphyletic and some monophyletic (Figure 2c)—as identified using mtDNA but with completely different phylogeographic relationships (Figures 1 and 2). In another *Anolis* species, mito‐nuclear discord was identified across two contact zones (Ng & Glor, 2011), where differential patterns of gene flow between mtDNA and nuclear DNA suggested sex‐biased dispersal in one transect, and a lack of nuclear DNA gene flow (i.e., partial reproductive isolation) and some mtDNA introgression across the other transect.

In contrast, we found the same genetic clusters using nuclear DNA loci (Figure 2) as those previously identified using mtDNA (Tollis & Boissinot, 2014), but the genetic groups differ in how they are related between datasets. Because of this pattern, it is unlikely that biased dispersal for one of the sexes or differential introgression caused the observed patterns. Alternatively, the observed pattern of different evolutionary relationships between marker types is likely due to stochastic lineage sorting of shared ancestral variation. Generally, large population sizes (Figure 5, Table S5) across a large possible range increase stochasticity of lineage sorting. Based on individual gene trees, only the southern Florida phylogeographic cluster is monophyletic for any loci (Figure 3c; GSI values equal to 1), indicating that the overall phylogeographic signal is from differential allele frequencies (i.e., partial lineage sorting) across loci. Indeed, in another analysis looking at discordance across gene trees (BUCKy), we find two consistent patterns: (1) southern Florida as basal to all other lineages of *A. carolinensis* and (2) the Carolinas and Gulf‐Atlantic lineages as sister to each other. Although BUCKy analyses found inconsistent results for two of the lineages—eastern and northwestern Florida—the unvarying results are in discordance with previous mitochondrial studies. However, in agreement with the GSI results, the concordance factors had wide confidence intervals suggesting lack of complete lineage sorting across most loci. These patterns could simply be due to shared ancestral polymorphisms in the period of transition from polyphyly to paraphyly to monophyly of each independently evolving lineage. An alternative is that some level of gene flow is precluding complete lineage sorting across all loci, although not enough to diminish the overall signatures of genetic structure between the lineages. Whether the lack of signal per locus is due to ascertainment bias of targeted loci or is simply because of genome‐wide retained shared ancestral variation remains to be determined (e.g., with RAD‐seq or genome resequencing data).

### 4.2 Biogeographic patterns and comparison with co‐distributed taxa

Vance (1991) described two subspecies in *A. carolinensis*:* A. c. seminolus* in southwestern Florida and *A. c. carolinensis* throughout the rest of the range. While this initially appears to line up with the split between the southern Florida phylogeographic cluster and all other *A. carolinensis* phylogeographic groups, Vance (1991) also described intergrades between the two subspecies ranging from southern Florida up into Alabama and Georgia; the observed genetic patterns are discordant with this described subspecific morphological variation.

In a review of the phylogeographic studies in unglaciated eastern North America—a region completely overlapping with the distribution of *A. carolinensis*—Soltis et al. (2006) surveyed patterns in ~150 species of plants and animals. This review included species of terrestrial vertebrates (e.g., mammals, reptiles, amphibians) that may be directly compared with our results here. However, the only consistent terrestrial phylogeographic breaks—and in contrast with those we found in *A. carolinensis*—are across three major rivers (Apalachicola, Mississippi, and Tombigbee) and the Appalachian Mountains (Soltis et al., 2006). Because these general phylogeographic patterns are not similar to the patterns observed here in *A. carolinensis*, below we discuss the few examples of phylogeographically concordant patterns, beginning with the oldest phylogeographic divergences.

The first major phylogeographic break observed here (Figure 2) is between southern Florida and more northern populations. This split is consistent with the switch between temperate conifer and flooded grasslands/savannas biomes (Wade, Riitters, Wickham, & Jones, 2003) and is a similar break to that found in white‐tailed deer (*Odocoileus virginianus*; Ellsworth et al., 1994); while our results are consistent with northward expansion out of southern Florida, based on phylogeographic and genetic diversity patterns (Figures 2c, 3a,b), white‐tailed deer were hypothesized to colonize southern Florida from more northern populations (Ellsworth et al., 1994). Additionally, while we estimated a late Miocene or Pliocene divergence of southern Florida populations (Table 3), the white‐tailed deer were hypothesized to diverge during Pleistocene interglacials (Ellsworth et al., 1994).

While the timing of white‐tailed deer divergence is incongruent with our results, the mechanism of diversification may be similar. During the Pliocene warm periods and Pleistocene interglacials, various archipelagos (at different time periods) experienced isolation from peninsular Florida (Petuch & Roberts, 2007), providing a mechanism for isolation and subsequent diversification. This scenario potentially explains the complex split between the eastern and northwestern Florida phylogeographic groups as well, albeit during the Pleistocene, because there are no obvious riverine or biogeographic barriers separating these populations. The east–west split in Florida is not found in other taxa, but does correspond to the general pattern of river drainages into the Gulf of Mexico or Atlantic Ocean.

More recently, there is the split in northern Florida between the Florida phylogeographic groups and the more northern Gulf‐Atlantic and Carolinas populations. While this region has not been explored as a major phylogeographic break between genetic lineages (e.g., Soltis et al., 2006), it is described as a suture zone between many subspecific forms (Remington, 1968), including reptiles, mammals, birds, invertebrates, and plants. This is suggestive of a widespread pattern across plants and animals due to similar mechanisms of diversification. The taxa identified by Remington (1968) are generally subspecific splits, and those observed here in *A. carolinensis* are between relatively recent genetic divergences (Pleistocene, Table 3), all suggestive of recent divergence via isolation in Pleistocene glacial maxima refugia or interglacial islands. More recently, using molecular methods, a shallow phylogeographic divergence was found in a widespread snake (*Agkistrodon piscivorus*) species (Guiher & Burbrink, 2008).

As mentioned above, the major concordant splits identified across terrestrial taxa are major riverine barriers and the Appalachian Mountains (Soltis et al., 2006). Here, we found no evidence that the Apalachicola, Mississippi, or Tombigbee Rivers preclude gene flow among populations in *A. carolinensis*. While the Savannah River—splitting populations in North and South Carolina from the rest of the Gulf‐Atlantic Clade—could explain one break, it may be a sampling artifact, as this phylogeographic break was identified in a different location by Campbell‐Staton et al. (2012; based on different sampling) and does not correspond with the riverine barrier. We identify numerous admixed individuals between the Gulf‐Atlantic and Carolinas clades occurring in the coastal plain regions of Georgia and South Carolina, with no evidence for allele sharing between individuals further north in eastern Tennessee and nearby South Carolina or North Carolina. This may indicate that elevational gradients, such as those occurring along the Appalachian and Piedmont plateaus and the Blue Ridge, have been more effective dispersal barriers during the mainland colonization of green anoles than riverine barriers.

## 5 Conclusion

*Anolis carolinensis* is an emerging model organism for biomedical studies with a complete genome sequence; it has a rich evolutionary history across a dynamic southeastern North American landscape that has experienced major topographic and climatic upheavals in the last few million years. With so much morphological, physiological, and genomic diversity observed across its range, the role of adaptation, drift, and phenotypic plasticity across different populations of *A. carolinensis* remains an open question. In addition, the effect of this natural variation on laboratory studies that include individuals collected from different regions will be an area ripe for investigation. Here, we provide a foundation for this future work by demonstrating a robust and well‐supported phylogeny of the five major green anole clades, using hundreds of DNA sequence markers. As in previous studies, we identify Florida as the origin for green anole diversity in North America, and populations in southern Florida as the sister lineage to the rest of the species. We find novel evidence for a step‐wise pattern of northward diversification out of Florida, a sister–group relationship between two major mainland clades, and gene flow between Florida and the mainland. The individual sequenced by the Broad Institute for the green anole genome project was collected in South Carolina (Alföldi et al., 2011); future resequencing efforts should reveal if it is indeed representative of the rest of the species in terms of genomic structure.

## Conflict of Interest

None declared.

## Data Accessibility

All data used for analyses are available in the Dryad Digital Repository (doi: 10.5061/dryad.tq0g0), including: (1) all trimmed sequence data alignments, (2) RAxML gene trees, multilocus bootstraps, and species trees, (3) concatenated sequence matrix and RAxML concatenated tree output, (4) TreeMix output tree and bootstraps, (5) SVDquartets input file and output bootstraps, (6) genealogical sorting index output, (7) extended Bayesian skyline plots xml input and EBSPAnalyser output, (8) G‐PhoCS input, control, and output files. Other outputs (e.g., STRUCTURE, DAPC) are available in the online supplementary information.
